# The Role of Oxidative Stress in Carcinogenesis Induced by Metals and Xenobiotics

**DOI:** 10.3390/cancers2020376

**Published:** 2010-04-08

**Authors:** Frank Henkler, Joep Brinkmann, Andreas Luch

**Affiliations:** German Federal Institute for Risk Assessment, Thielallee 88-92, 14195 Berlin, Germany; E-Mail: Joseph.Brinkmann@bfr.bund.de

**Keywords:** metals, xenobiotics, ROS, oxidative stress, carcinogenesis

## Abstract

In addition to a wide range of adverse effects on human health, toxic metals such as cadmium, arsenic and nickel can also promote carcinogenesis. The toxicological properties of these metals are partly related to generation of reactive oxygen species (ROS) that can induce DNA damage and trigger redox-dependent transcription factors. The precise mechanisms that induce oxidative stress are not fully understood. Further, it is not yet known whether chronic exposures to low doses of arsenic, cadmium or other metals are sufficient to induce mutations *in vivo*, leading to DNA repair responses and/or tumorigenesis. Oxidative stress can also be induced by environmental xenobiotics, when certain metabolites are generated that lead to the continuous release of superoxide, as long as the capacity to reduce the resulting dions (quinones) into hydroquinones is maintained. However, the specific significance of superoxide-dependent pathways to carcinogenesis is often difficult to address, because formation of DNA adducts by mutagenic metabolites can occur in parallel. Here, we will review both mechanisms and toxicological consequences of oxidative stress triggered by metals and dietary or environmental pollutants in general. Besides causing DNA damage, ROS may further induce multiple intracellular signaling pathways, notably NF-κB, JNK/SAPK/p38, as well as Erk/MAPK. These signaling routes can lead to transcriptional induction of target genes that could promote proliferation or confer apoptosis resistance to exposed cells. The significance of these additional modes depends on tissue, cell-type and is often masked by alternate oncogenic mechanisms being activated in parallel.

## 1. Introduction

The group of reactive oxygen species (ROS) include superoxide anion radical (O_2_^•−^), hydrogen peroxide (H_2_O_2_) and hydroxy radical (•OH) molecules that are generated by consecutive intracellular reduction of molecular oxygen. O_2_^•−^ is mainly generated as a side-product of mitochondrial respiration, when electrons are transferred by ubiquinone or semi-ubiquinone directly to oxygen instead of successive acceptors in the respiratory electron transfer chain [1]. 

Such side reactions can also occur at the iron-sulfur components of complex I and III. It is estimated that up-to 5% of total oxygen consumed by mitochrondria is converted into the superoxide anion radical [2]. O_2_^•−^ is also generated by NADPH oxidases of phagocytes. Other considerable endogenous sources include metabolizing enzymes such as 5-lipoxygenase, xanthine oxidase, and to a lesser extend the cytochrome P450-dependent monooxygenases (CYPs) [3]. Compared to the mitochondrial redox-systems, an accidental electron transfer from CYPs to oxygen is less frequent, because no intermeditate electron carriers are released from CYP enzymes. O_2_^•−^ can be regarded as the principle ROS, because it represents one reduction equivalent only. This molecule is short-lived and limited in its capacity to pass cellular membranes. The major proportion of O_2_^•−^ is disposed into hydrogen peroxide (H_2_O_2_) and molecular oxygen by superoxide-dismutase (SOD):

2 O_2_^•−^ → H_2_O_2_ + O_2_

Hydrogen peroxide is fairly stable, capable of passing cellular membranes and can thus be regarded as the central ROS in carcinogenesis. It is disposed by catalases and gluthatione peroxidases into oxygen and water. Importantly, H_2_O_2_ is a strong oxidant that can itself further be reduced to the hydroxy radical (•OH). This can occur in a Haber-Weiss reaction by oxidation of superoxide anion radical:

O_2_^•−^ + H_2_O_2_ → •OH + OH^—^ + O_2_

Although this reaction is slow, it is catalyzed by iron. In fact, Fe^2+^ can also directly reduce hydrogen peroxide *via* Fenton’s reaction, generating hydroxy radicals as well:

Fe^2+ ^+ H_2_O_2_ → Fe^3+ ^+ •OH + OH^−^

However, reduction of O_2_^•−^ by SOD or non-enzymatic mechanisms is not the major pathway of H_2_O_2 _generation. It has long been known that up-to 80% of H_2_O_2_ is formed by peroxisomal and microsomal enzymes [4]. For example, peroxisomes generate a major proportion of H_2_O_2 _during β-oxidation of long-chain fatty acids. The biochemistry of peroxisomal β-oxidation differs from its mitochondrial counterpart as acyl-CoA oxidase triggers the initial step, thereby generating *trans*-2,3-dehydroacyl-CoA along with H_2_O_2_ [5,6]. The former compound is subsequently degraded into acetyl-CoA units by consecutive oxidation cycles. Hydrogen peroxide is also a side-product of other peroxisomal oxidases, such as D-amino acid oxidase, D-aspartate oxidase or polyamine oxidase, respectively [7]. The overall activity of peroxisomal enzymes together may account for up-to 20% of the total cellular oxygen consumption in liver cells [5]. Microsomal CYP-mediated ω-oxidation of fatty acids is also discussed as an important route for H_2_O_2_ formation [8].

Hydroxy radicals are highly reactive, but short-lived molecules that trigger DNA damage. DNA modifications resulting from these radicals, especially 8-hydroxy 2’-deoxyguanosine (8-OHdG), may give rise to mutations when DNA repair systems are overloaded or compromised. Mutagenesis triggered by •OH is a major factor contributing to the carcinogenic risk related to conditions of increased oxidative stress. In fact, the urinary 8-OHdG level is regarded as important biomarker for oxidative DNA injuries in animal models and human patients alike [9]. Moreover, •OH can also react with other cellular molecules, thereby denaturing enzymes or structural proteins or initiating peroxidation of polyunsaturated fatty acids. The latter process can trigger the degradation of phospholipids and impair membranous cellular structures, thereby contributing to acute toxic effects of some compounds discussed below. 

Metalloid compounds and xenobiotics are known to induce carcinogenesis. For instance, clinical investigations have illustrated that a disturbed homeostasis of intracellular iron is related to an increased risk for cancer [10]. Hereditary hemochromatosis is a metabolic disease associated with iron overload mainly in liver and constitutes a major risk factor for hepatocellular carcinoma in developed countries [11]. Induction of oxidative stress is regarded as important mechanism underlying the carcinogenic risk associated with abundant iron levels [10].

One central mechanism that regulates lipid metabolism and asserts a major impact on endogenous ROS levels is controlled by peroxisome proliferator-activated receptors (PPARs) [8]. PPARα is a key regulator of fatty acid oxidation and typically activated by lipids and long-chain fatty acids that undergo mircrosomal or peroxisomal degradation [12]. However, organic solvents, pharmaceuticals, such as fibrate drugs, certain phthalates (industrial compounds that are widely used as plasticizers in soft PVC), as well as other synthetic materials can replace endogenous ligands. Target genes of PPARα include acyl-CoA oxidase, as well as CYP4A1 and 4A6. Sustained PPAR activation results in elevated H_2_O_2_ levels and oxidative stress [12]. Further a crucial role of sustained PPARα activation in liver carcinogenesis was demonstrated in acyl-CoA oxidase “knock-out” mice [13]. Xenobiotic activators of PPARs thus need to be considered as potential nongenotoxic carcinogens, at least in rodents. Intriguingly, activity levels of PPAR agonists display wide variations depending on species, a fact that limits conclusions drawn from animal experiments. For example, di-(2-ethylhexyl)phthalate (DEHP) clearly acts as a PPAR activator in rodents, but apparently not in human cells. However, this substance remains problematic for human health, because mechanisms that trigger adverse effects have not yet been defined in detail [14].

In this minireview, we will discuss the relevance of oxidative stress, generated by metals, metalloid compounds or xenobiotics for carcinogenesis. Firstly, we will summarize the evidence for occurrence of oxidative stress at exposure levels that are relevant for human health. Secondly, we discuss the specific significance of ROS for the toxicity of these ions or organic compounds. However, considerable uncertainties apply for the latter issue since most compounds included in our examination concurrently affect multiple genotoxic and nongenotoxic mechanisms and thus warrant further investigation. 

At the inorganic side we have focused our discussion on arsenic, chromium, nickel and cadmium, because these species (or selected compounds thereof) are classified as category 1 carcinogens (“carcinogenic to humans”), according to the International Agency for Research on Cancer (IARC, see: http://monographs.iarc.fr/ENG/Classification/index.php) and the German Commission for the Investigation of Health Hazards of Chemical Compounds in the Work Area (“MAK” commission) [15].

## 2. Carcinogenesis and Oxidative Stress Associated with Selected Metals and Metalloids

### 2.1. Arsenic (As)

Arsenic is one of the most important toxic metals and is classified as an IARC category 1 carcinogen. Human exposure can occur through contaminated drinking water and constitutes a serious health problem in parts of India and Bangladesh. Other important sources include food, especially rice, cereals or seafood [16]. In 2009, investigations in Germany pointed to elevated levels of arsenic in rice waffles, marketed for nutrition of small children [17]. Arsenic contamination of drinking water has been epidemiologically linked to increased mortality from lung and bladder cancer [18], as well as cardiovascular diseases [19]. Inorganic arsenic [As(V), As(III)] is efficiently absorbed in the intestine and converted into methylated species. In fact, methylated metabolites such as monomethyl (MMA) and dimethyl arsonous acid (DMA) trigger genotoxic effects similar to inorganic arsenite [20]. 

**Figure 1:**
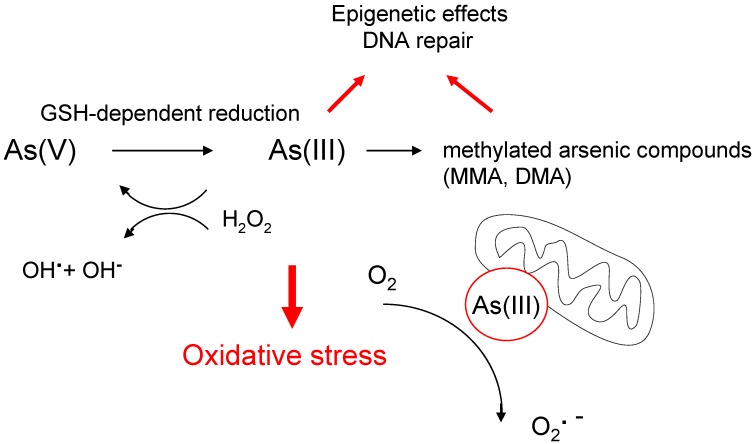
Carcinogenic mechanisms of arsenic compounds. Inorganic arsenic compounds and methylated metabolites display similar genotoxic properties. Generation of oxidative stress is regarded as central mechanism in As-mediated carcinogenesis. However, the precise mechanisms of ROS-formation are not yet clarified. A mitochondria-dependent mechanism and a H_2_O_2_/hydroxy radical pathway are discussed. In addition, arsenic affects DNA methylation and DNA repair enzymes (see text for details).

Multiple mechanisms have been suggested to contribute in arsenic induced carcinogenesis (Figure 1). Besides its recognized capacity to induce oxidative stress [21], arsenic also interacts with cellular targets such as the thiol groups of various proteins. In fact, *S*-adenosyl methionine (SAM) and glutathione (GSH) are required at several stages for metabolic conversion of both arsenite [As(III)] and arsenate [As(V)]. The capacity of trivalent arsenic to bind thiol groups has been suggested as trigger for inactivation of various zinc-finger proteins [22]. Recent *in vitro* studies demonstrated that potential targets include DNA repair enzymes such as XPA and XPD [22]. Arsenic was further shown to inhibit nuclear excision repair (NER) of DNA adducts caused by other genotoxins, as for example benzo[*a*]pyrene [23] and to act as co-carcinogen in concert with other mutagens [24]. Although no arsenic compound has been shown to directly form covalent DNA adducts, accumulation of DNA damage triggered by oxidative stress might be enhanced through concomitant inhibition of repair pathways. 

Inorganic arsenic compounds are also known to interact with methyltransferses and are substrates of arsenite methyltransferase AS3MT. This association might link arsenic with epigenetic mechanisms of gene expression regulation. In fact, chronic exposure to inorganic arsenic has been proposed to cause hypomethylation of DNA, thereby enhancing the expression of estrogen receptor-α (ERα) and cyclin D1 [25]. Both proteins promote cell cycle progression and might thus contribute to an increased oncogenic risk. Methylation of arsenite could possibly lead to depletion of SAM and therefore account for activation of another set of genes involved in C1 (methyl) metabolism. Intriguingly, DNA hypermethylation was also reported and shown to inhibit expression of tumor suppressor proteins, especially p53 and the cyclin dependent kinase inhibitor p16^Ink4a^ [26,27,28]. Regarding these different effects on individual genes, the overall relevance of modified methylation patterns and their relevance for tumorigenesis remain elusive. 

Induction of oxidative stress has been proposed as a major mode of action in arsenic induced carcinogenesis [29]. The occurrence of oxidative stress in cells and tissues and the resulting increased carcinogenic risk in arsenic-exposed people have been established, but grossly rely on the detection of biomarkers, especially 8-OHdG in urine [30]. However, the precise mechanisms of ROS generation have not yet been clarified, but might involve formation of hydroxy radicals [31]. Some lines of evidence suggest that mitochondria are the primary target. Arsenic triggers rapid morphologic changes in this organelle and leads to inactivation of mitochondrial enzymes and loss of mitochondrial membrane potential. It has been suggested that arsenite constitutes a bypass for electrons from the respiratory chain, thereby facilitating the formation of superoxide anion radical [32]. Additional proposed mechanisms include the reduction of oxygen by As(III), thereby leading directly to the generation of H_2_O_2 _and/or formation of arsenic peroxyl radicals as central mediators of DNA damage [33]. It remains difficult to clarify the individual significance of each of the potential toxic mechanisms of arsenic, although there is growing consensus regarding a predominant role for ROS generation. Arsenic compounds have also been shown to activate transcription factor AP-1 and nuclear factor NF-κB [34,35], which both are key proteins contributing to cell proliferation regulation. NF-κB exerts oncogenic effects when permanently activated. ROS-dependent alterations in the activity of transcription factors could also enhance proliferation and possibly promote both accumulation of mutations and carcinogenesis in exposed cells [29]. Intriguingly, in respect to NF-κB an important exception was observed in epithelial cells of the lung. When treated with arsenite, these cells displayed a ROS-dependent inhibition of NF-κB, presumably because of oxidation of cysteine 179 in the inhibitor of NF-κB kinase (IKK) β [36]. 

### 2.2. Chromium (Cr)

Association of chromium with maligant diseases, especially lung cancer has first been recognized as an occupational health hazard in industrial branches like steel wielding, tanneries or chromium plating [37]. Areas of consumer exposure include leather textiles, exhaust from cars or waste disposal, as well as cigarette smoke. A recent study also suggested chromium as risk factor for breast cancer [38,39].

Cr(VI) compounds (e.g., CrO_4_^2–^) have been classified as human carcinogens by the IARC. In contrast to Cr(III), negatively charged chromate ions (CrO_4_^2–^) can efficiently penetrate anionic channels in cellular membranes, followed by intracellular reduction to Cr(V) and Cr(III) compounds. These sequential reductions occur after Cr(VI) is bound by GSH [40,41], but GSH can be replaced by other cellular reductants, as for example ascorbate. In contrast to Cr(VI), Cr(V) and Cr(III) compounds can directly interact with DNA, thereby forming binary chromium-DNA adducts, or cross-links between DNA and proteins, ascorbate or gluthathione, respectively [42]. Although Cr(III) is not considered a human carcinogen, it plays an apparent key role in the carcinogenesis triggered by hexavalent chromium. The differences in toxicological properties of various chromium ions are most likely related to the limited capacity of Cr(III) to enter mammalian cells. Transformation and transfection experiments in bacteria [43] and human fibroblasts [44] proved mutagenicity of trivalent chromium once it has reached the intracellular compartment.

**Figure 2:**
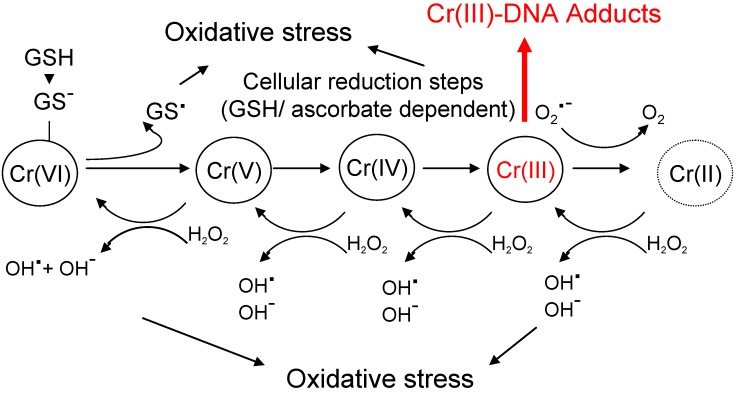
Carcinogenic mechanisms of chromium compounds. Chromium (VI) compounds are internalized in cells *via* anionic channels. Cr(VI) is then reduced and accumulates as trivalent ion. Formation of Cr(III)-DNA adducts is regarded as predominant carcinogenic mechanism (see text for details). In parallel, chromium ions can engage in Fenton-like reactions, generating hydroxy radicals. However, molecular details of these reactions need still to be clarified. The overall relevance of oxidative stress for chromium mediated carcinogenesis remains controversial.

Besides DNA adduct formation *via* Cr(III), exposure to Cr(VI) can also trigger the generation of ROS and oxidative stress, which had been previously shown to promote chromium-induced DNA-damage [45]. Again, several mechanisms have been suggested (Figure 2). For example, reduction of Cr(VI) generates gluthathione-thiyl radicals [46] that can reduce molecular oxygen to superoxide anion radicals. Both Cr(IV) and Cr(III) can also participate in Fenton-type reactions that generate hydroxy radicals [47]. Notably, these Fenton reactions occur in parallel to the reduction of chromium and reconvert the compound into higher oxidation states. The induction of futile redox-cycles is therefore feasible. Chromium-mediated generation of hydroxy radicals can furthermore occur by Haber-Weiss reactions, which depend on endogenous superoxide anion radical and H_2_O_2_ [47]. Although mechanisms of chromium-induced oxidative stress are well-studied *in vitro* and in cells in culture, the overall relevance for carcinogenesis is still a matter of debate. Experiments by Ye and co-workers [48] confirmed the generation of hydroxy radicals in cells treated with Cr(VI). However, •OH generation was only detectable at concentrations that also triggered severe cytotoxicity. This study may argue for a minor contribution of ROS and rather supports a predominant role of DNA adducts in chromium-induced carcinogenesis [15]. Further studies are required for clarification.

### 2.3. Nickel (Ni)

Nickel is among the most important human allergens, but also classified as human carcinogen. Nickel-carbonyl vapours and other sources of inhalation exposure have been identified as occupational risk for developing lung cancer [37,49]. The carcinogenic effects of inhalative nickel exposure have been confirmed in animal experiments [50]. Tumorigenic properties of the metal are partly related to the generation of ROS and the disturbance of intracellular redox homeostasis is implied.

Ni(II) ions have been shown to trigger DNA hydroxylation as well as deglycosylation of dG residues [51]. Oxidative DNA damage further included intrastrand DNA cross links, double strand breaks and formation of 8-OHdG [52]. In lymphocytes, nickel compounds induced sister chromatid exchanges, which were clearly attributed to oxidative stress [53]. 

Although oxidative stress is a recognized factor in the carcinogenesis of nickel [54] uncertainties remain about required dosage and exposure levels that are sufficient to generate relevant amounts of ROS. In this regard, wide variations have been observed between different cell lines [15]. As for other carcinogenic metals, alternate mechanisms of tumorigenesis are discussed for nickel as well. There are some similarities with arsenic, since nickel sulfide can also decrease DNA methylation [55], On the other hand, nickel was also shown to trigger hypermethylation of p16^Ink4a^ and to inhibit the expression of this tumor suppressor protein in response to oxidative stress [56]. Interestingly, suppression of p16^Ink4a^ has recently been proposed as common mechanism in ROS-mediated carcinogenesis [57] and therefore could play a central role in the chronic toxicity of metals. 

In addition, Ni(II) is further known to inhibit various DNA repair mechanisms [58] and acts as powerful co-mutagen for genotoxic stimuli, such as UV-radiation [59]. The ion was further reported to induce gene silencing by interacting with chromatin and inhibition of histone acetylation [60,61,62]. 

Nickel is a potent inducer of hypoxia inducible factor-1α (HIF-1α) activity, too [63]. Under normoxic conditions, HIF-1α, which is the key transcription factor in regulating cellular responses to reduced oxygen pressure, is hydroxylated *via* prolyl-4 hydroxylase domain proteins (PHD1-3). PHDs are considered to participate in the cellular oxygen sensing system. The reaction catalyzed by PHDs depends on intracellular levels of oxygen, Fe^2+^ and 2-oxoglutarate. Hydroxylation targets HIF-1α for proteasomal degradation through its binding to the von Hippel-Lindau (VHL) protein [64]. PHD activity is inhibited under hypoxic conditions resulting in stabilized HIF-1α. HIF-1α dimerizes with ARNT to form the transcription factor HIF-1. Major target genes include glycolytic enzymes, erythropoietin [65], as well as regulators of angiogenesis such as vascular endothelial growth factor [66]. The capacity of HIF-1 signaling to promote carcinogenesis may be related to the activation of genes encoding anti-apoptotic (Bcl2) and multi drug resistance (MDR) proteins [67]. The mechanism of Ni-mediated induction of HIF-1 is as yet not completely understood, but likely to involve the inhibition of PHD enzymes and the block of Fe^2+^ delivery into cells (for a review, see [68]). 

**Figure 3:**
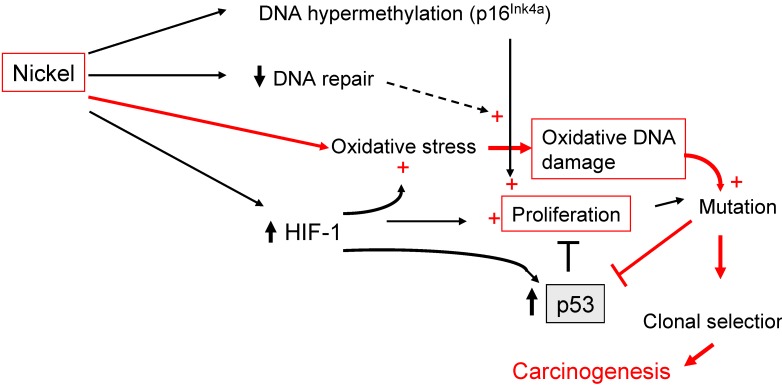
Carcinogenic mechanisms of nickel. Nickel ions can induce oxidative stress, which provides a primary genotoxic stimulus required for carcinogenesis (red lines). In addition, Ni(II) triggers multiple mechanisms that can amplify the moderate effects of oxidative stress (plus sign, 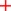). An interplay of enhanced proliferation and up-regulation of p53 could constitute a strong selective pressure, favouring mutations, which may inactivate tumor suppressor genes (see text for details).

A central role for HIF-1 in nickel carcinogenesis has been suggested by experiments in mouse embryonic fibroblasts deficient for HIF-1. These cells did not display an increment in soft agar growth upon nickel exposure, in contrast to wild-type cells [69]. The weak genotoxic effects of nickel that primarily originate from oxidative stress may be amplified by both epigenetic modifications and HIF-1 signaling. One potential mechanism could also involve the tumor suppressor protein p53, which is induced and stabilized by both hypoxia [70] and intracellular nickel [71]. However, p53 inhibits the cellular capacity to respond to hypoxia [72]. Whereas HIF-1 stimulates cell proliferation, p53 and other tumor suppressor proteins trigger antagonizing effects, such as growth arrest or apoptosis. This might lead to a strong selective pressure that favors cell populations with accumulated mutations in tumor suppressor genes (Figure 3). Moreover, the activation of HIF-1 signaling by nickel is likely to induce vascularization of growing tumors.

### 2.4. Cadmium (Cd)

Major routes of exposure to this toxic heavy metal include occupational sources, cigarette smoke and food, especially seafood, mushrooms and chocolate [73]. Cd(II) is sequestered by metallothionin, accumulates in liver and kidney and the biological half-life of renal Cd is up-to 30 years. Proximal tubule cells are the main cellular targets of Cd-mediated nephrotoxicity. Besides acute and chronic kidney damage, Cd(II) is further classified as carcinogen [74] triggering tumors in lung, kidney and prostate [75]. The mechanisms of carcinogenesis are far from being completely clarified, but might involve the replacement of essential metals in various biomolecules and enzymes. For example, replacement of zinc in zinc finger structures was proposed as molecular basis for the inactivation of DNA repair enzymes, including XPA [76]. Cadmium was further shown to selectively inhibit 8-oxo-dGTPase, an enzyme that hydrolyzes mutagenic oxidation products of dGTP species [77]. Notably, adverse effects on DNA repair are already apparent at low or moderate exposure levels. Cadmium inhibited base excision repair (BER) at concentrations that were not sufficient to induce ROS [58]. Mechanisms contributing in NER or mismatch repair (MMR) are sensitive targets for cadmium and regarded as major target for cadmium induced carcinogenesis [78]. 

Multiple studies have demonstrated that cadmium can affect cellular redox homeostasis [79]. Analysis of tumors formed from Cd-exposed 3T3 cells in nude mice revealed increased cellular levels of superoxide anion radical and hydrogen peroxide, concomitant with the up-regulation of proto-oncogenes, especially c-fos, c-jun and c-myc [80]. Cadmium-induced ROS were furthermore shown to trigger genotoxicity, including DNA double strand breaks in mammalian cells [81]. ROS generation could thus contribute to the carcinogenic potency of cadmium, but, nevertheless also triggers additional effects, including apoptosis [82]. Cadmium-induced ROS are further considered as important hallmark of acute toxicity and capable of inducing lipid peroxidation and inflammation in lung tissue of animals [83]. 

**Figure 4:**
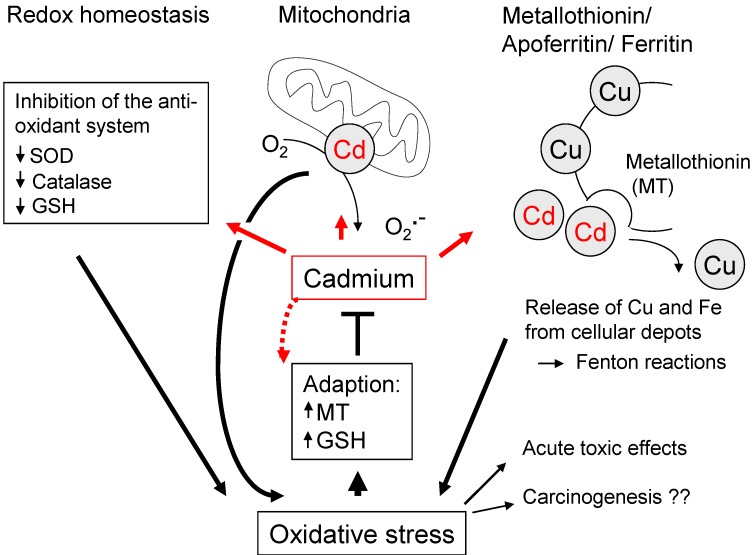
Cadmium and oxidative stress. Cadmium does not belong to redox-active metals. Several mechanisms for generation of ROS have been proposed though (see text for details). Chronic Cd(II) exposure can also induce expression of metallothionin (MT) and triggers adaption mechanisms towards oxidative stress, thus limiting the role of ROS in carcinogenesis. Alternate carcinogenic mechanisms of cadmium, such as inhibition of DNA repair, are not shown in this illustration.

Since Cd(II) is not redox-active, replacement of iron and copper ions from intracellular depots, especially ferritin and apoferritin [84], has been discussed as indirect source for oxidative stress. Notably, this concept is supported by experiments demonstrating the generation of hydroxy radicals by cadmium ions in the presence of copper reconstituted metallothionin [83]. In addition, cadmium was shown to inhibit complex III of the mitochondrial respiratory chain (Figure 4). This alternative route of ROS generation leads to the accumulation of semiubiquinones and the formation of superoxide anion radical [86]. 

Another aspect comes from the suppression of the cellular anti-oxidant system by cadmium, as an indirect trigger of oxidative stress (Figure 4). For example, cadmium inhibits expression of antioxidant enzymes such as SOD and catalase [87], thereby contributing to augmented levels of O_2_^•−^ and H_2_O_2_ and subsequent lipid peroxidation. On the other hand, Cd(II) had been shown to deplete GSH in rat liver and kidney cells, possibly by activating γ-glutamyl transpeptidase (γ-GT) or by depleting NADPH secondary to reduction of oxidized lipids [88]. In addition, ROS can be generated *via* cytokines, because of pro-inflammatory effects of Cd(II) in liver tissue [89]. 

ROS are implicated in the toxicology of cadmium, mainly *via* the peroxidation of lipids. However, the significance for carcinogenesis remains controversial [90], partly because of conflicting data on ROS levels in Cd-induced tumors. In fact, transformation of human urothelial cells by cadmium occurred already at low concentrations, insufficient to trigger ROS, but not at higher exposure levels causing oxidative stress [91]. Because accumulation of ROS is not stringently associated with carcinogenesis or chronic toxic effects, only a minor role was suggested for oxygen species in malignant transformation by cadmium [90]. In contrast, inhibition of DNA repair, epigenetic alterations of DNA methylation [92] and resistance towards apoptosis [93] have been proposed as dominant mechanisms in Cd-mediated carcinogenesis. The reason for the limited impact of ROS might lie in the adaption of long-term cadmium-exposed cells by up-regulation of antioxidant proteins. Elevated levels of GSH [94], as well as antioxidant enzymes and metallothionein have been observed [95].

## 3. Oxidative Stress Associated with Organic Compounds—Implications for Carcinogenesis

An important mechanism of ROS generation by carcinogenic xenobiotics, as for example polycyclic aromatic hydrocarbons (PAHs), involves conversion of these substances into quinones. This occurs primarily by oxidation into phenolic intermediates that can be further converted *via* semiquinone anion radicals into *ortho*-quinones [96]. H_2_O_2_ and superoxid anion radicals (O_2_^•−^) are generated in this process. Importantly, quinones are substrates of various reductases, such as NAD(P)H: quinone oxidoreductase (NQO1) [97]. As long as the reducing capacity of cells is maintained, these compounds can be converted back to hydroquinones or catechols, which then might undergo auto-oxidation again to constantly generate H_2_O_2_ and O_2_^•−^ *via* futile redox-cycling [98]. This process is regarded as major source for ROS in cells exposed to PAHs. 

Benzo[*a*]pyrene (BP) is an important example. Like other PAHs, this substance is metabolized by various alternate routes. The generation of the BP-7,8-diol-9,10-epoxide (BPDE) is regarded as the predominant mechanism for carcinogenesis, as this metabolite can potently react with guanine or adenine residues to form bulky DNA adducts. In addition to this pathway, the specific impact of BP quinone formation (e.g., 1,6-BPQ and 3,6-BPQ) and subsequent generation of ROS [99] on potential oncogenic pathways has been investigated particularly in breast epithelial cells [100]. In these experiments, BPQ-triggered ROS were associated with activation of EGFR (epidermal growth factor receptor), leading to enhanced proliferation. However, in a more recent study, this effect was partly attributed to activation of the arylhydrocarbon receptor (AhR), which was induced in parallel by both BPQ metabolites [101]. There is further evidence that BP enhances H_2_O_2_ formation synergistically with UVA-radiation and promotes formation of 8-OHdG lesions in DNA of epidermal cells [102]. In HepG2 cells, BP triggered an antioxidant response, including elevated levels of GSH [103]. The role of ROS in BP-induced carcinogenesis, however, remains obscure. Due to the dominance of genotoxic mechanisms resulting in the formation of bulky DNA adducts, ROS-mediated subtle structural damage or impairment of cellular signaling becomes elusive and hard to be sorted out. 

TCDD (2,3,7,8,-tetrachlorodibenzo-*p*-dioxin) is an environmental pollutant, which is classified as human carcinogen. Like BP, TCDD is a strong agonist of AhR. After activation, this receptor triggers induction of multiple genes, involved in metabolism of xenobiotics. Notably, permanent activation of AhR is sufficient to induce spontaneous stomach tumors in mice [104], suggesting that oncogenic effects of TCDD depend on this receptor. Induction of CYP1 enzymes has been proposed as a mechanism leading to ROS generation, consecutive oxidative DNA damage and eventually tumorigenesis [105], for review, [106]. The capacity of TCDD to induce wide spectra of tumors in animals of both sexes has been well established [107]. Interestingly, in Sprague-Dawley rats, TCDD-induced liver tumorigenesis was significantly pronounced in female animals and depended on oxidative stress [108]. Further experiments suggested that ROS generation occurred after initial CYP-dependent oxidation of 17β-estradiol. This oxidation might amplify oxidative DNA damage, because catechol estrogenes can engage in redox-cycling mechanisms [109]. However, a predominant role of ROS-mediated mutations has been questioned in another animal study. Independent from gender, TCDD neither altered mutation frequency nor patterns in a transgene at concentrations where ROS were generated [110]. Since TCDD is not mutagenic, it was argued that alternate non-genotoxic pathways might account for carcinogenesis by altering gene expression [106]. The importance of AhR is emphasized by its central role in the disposal of xenobiotics and its activation by multiple ligands, which include selected PAHs, polychlorinated biphenyls, dibenzo-*p*-dioxins and dibenzofurans. Permanent active AhR signaling is clearly associated with an enhanced oncogenic risk. However, besides CYP, this receptor/transcription factor also induces expression of multiple other target genes such as c-ras or c-fos, thereby promoting inflammation and stress related signaling [111]. Similar target genes can be induced by ROS and such signaling effects could therefore contribute to carcinogenesis, even in the absence of detectable oxidative DNA damage. In fact, toxic metals, as well as TCDD [112] have been identified as powerful tumor promoters. Again, the specific contributions of ROS associated signaling remain to be elucidated in detail. 

## 4. Endogenous ROS Signalling and Tumor Promotion

**Figure 5:**
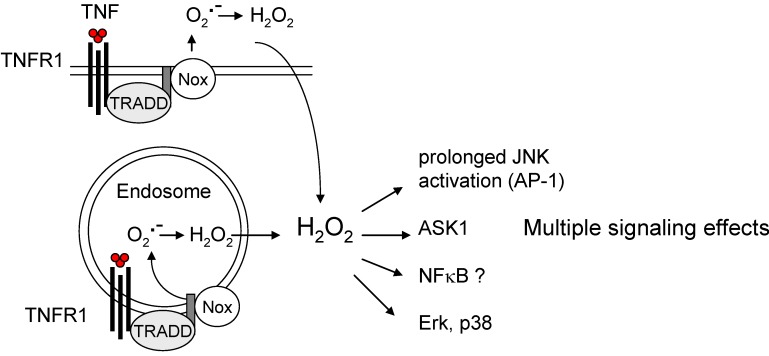
Role of endogenous ROS. Endogenous ROS are second messengers that are utilized by cytokine receptors, as for example tumor necrosis factor (TNF) α receptor 1 (TNFR1) (see text for explanation). The alternate ROS pathway, involving FAN and other signaling activities of TNFR1 are not shown in this illustration. TRADD, TNFR1-associated death domain protein; ASK1, apoptosis signal-regulating kinase 1.

ROS do not necessarily trigger adverse effects or constitute potential health risks. There is ample evidence that some of these molecules are integrated in signalling networks, utilized by cells to maintain redox homeostasis or to respond to oxidative stress. A gradual model has been proposed: low levels of ROS primarily activate the transcription factor Nrf-2, which induces expression of antioxidant enzymes. Increasing levels of oxidative stress also lead to the activation of transcription factors such as NF-κB and AP-1. Excessive oxidative stress perturbs the respiratory electron chain and triggers mitochondrial pore transition [113]. In this context, it should be emphasized that ROS are not only by-products or intermediates of oxidative metabolism, but also second messengers that are induced by specific signals to trigger well-defined down-stream effects. One important example is the interleukin receptor 1 (IL-R1). After cytokine-dependent activation, IL-R1 internalizes and recruits Rac1. This facilitates activation of Nox2 (NADPH oxidase 2), a membrane-bound oxidase, and the generation of O_2_^•−^. Within endosomes, superoxide anion radicals are converted into H_2_O_2_, which then activates the IL-R1-bound signalling complex and, as consequence, NF-κB signalling at the cytoplasmic side [114]. A similar mechanism applies for toll-like receptor 4 (TLR4), which plays an essential role in innate immunity. Like IL-R1, tumor necrosis factor (TNF) α receptor 1 (TNFR1) does also induce Nox-dependent generation of O_2_^•−^. The molecular details have recently been defined [115]. Although the relevance of this pathway for NF-κB activation *via* TNFR1 has been questioned based on inhibitor studies, ROS are involved in the TNFα-mediated prolonged activation of Jun N-terminal kinase (JNK) (Figure 5). Furthermore, an alternate mechanism depending on FAN (factor associated with neutral sphingomyelinase activation) leads to permeabilization of lysosomes and mitochondrial dysfunction [116] and is discussed to trigger a TNFα-mediated cytosolic accumulation of ROS. There is also evidence that TNFα signaling involves ROS-dependent modulation of histone acetyltransferases (HAT) and deacetylases (HDAC) [117]. The precise mechanisms of how ROS activate NF-κB are not understood. For AP-1, it has been demonstrated that ROS-dependent activation occurs through inactivation of phosphatases acting upstream of JNK [118]. 

It is temping to speculate that exogenous ROS and/or ROS generated by metals and xenobiotics may excessively increase endogenous ROS levels, thereby disturbing physiological signalling. This scenario is possibly relevant for low levels of chronic exposures that are insufficient to trigger acute toxicities, including apoptosis. ROS could contribute to carcinogenesis by influencing NF-κB, since this transcription factor promotes proliferation and angiogenesis. Furthermore, this redox-sensitive factor confers resistance towards apoptosis *via* induction of c-Flip and XIAP. Other target genes of NF-κB include manganese-dependent SOD (MnSOD) and key inflammatory cytokines, such as IL-1, IL-6 and TNFα, respectively [119]. 

AP-1 activates the expression of c-fos and ATF2, which promote proliferation and might thereby contribute to the tumorigenic effects of oxidative stress. However, SAPK/JNK signalling, which activates AP1 can also enhance apoptotic stimuli, thus antagonizing proliferative and proinflammatory signals at the same time. Importantly, ROS activate apoptosis signal-regulating kinase 1 (ASK1), which causes mitochondrial cytochrome c release and activation of effector caspases [120]. In TNFα signaling, ROS contribute predominantly to a prolonged activation of JNK/AP-1, which has been shown to primarily promote apoptosis, rather then proliferation [115]. The capacity of ROS to activate signaling is not limited to NF-κB and AP-1, but also includes MAP kinases (p38, Erk) and Akt. The down-stream effects of ROS signaling depend on both cell-type and cellular condition. The implications for carcinogenesis are therefore variable as well. 

## 5. Conclusions and Outlook

In this article, we have summarized the evidence that ROS contribute to carcinogenesis associated with the exposure to toxic metals or xenobiotics. Our discussion is focused on selected classified category 1 carcinogens, according to the IARC and the German MAK commission [15], whereas other metals, such as vanadium, lead and cobalt have not been considered. Although the latter compounds generate oxygen radicals in Fenton-type reactions and are able to cause oxidative DNA damage [15,121], their relevance for human carcinogenesis is much less clear. Cobalt is a highly interesting example, since it is an essential metal and part of the vitamin B_12_ complex. The capacity of cobalt to generate genotoxic radicals is strongly enhanced in combination with tungsten carbide (*i.e.*, wolfram carbide) [122] and, under such defined conditions, associated with an increased occupational risk for developing lung cancer [123].

Generation of oxidative stress is regarded as oncogenic risk factor because of two major effects. Firstly, occurrence of hydroxy radicals may lead to oxidative DNA damage. Secondly, a continuous disturbance of redox homeostasis can be associated with chronic pro-inflammatory signaling, leading to induction of proto-oncogenes and/or anti-apoptotic factors. Although a contributing role of ROS to carcinogenesis is widely acknowledged, putative adverse effects are frequently masked by alternate genotoxic or epigenetic mechanisms being triggered in parallel. In addition, adverse effects of radicals are balanced by adaption of the cellular anti-oxidant response, as discussed above for cadmium. This might explain experimental difficulties to specifically address the significance of ROS in carcinogenesis triggered by toxic metals or xenobiotics. 

Since oxidative stress is a relevant risk factor it needs to be carefully considered, especially in risk assessment of nanomaterials that have emerged in recent years. Although the number of applications is still limited, it is expected to grow rapidly in the years to come. Depending on material and surface properties, nanoparticles can generate ROS and essential issues, such as penetration into skin, internalization by mammalian cells, redox-properties or release of metal ions need to be addressed for a growing number of novel materials. The capacity of ROS to trigger and to promote carcinogenesis is therefore also of major concern for the emerging field of nanotoxicology. 
